# Non-familial Juvenile Polyposis Syndrome Presenting as Rectal Prolapse: An Unusual Presentation of a Rare Disease

**DOI:** 10.7759/cureus.11222

**Published:** 2020-10-28

**Authors:** Talal Almas, Salman Hussain, Reema Alsufyani, Hasan Alaeddin, Muhammad Kashif Khan

**Affiliations:** 1 Internal Medicine, Royal College of Surgeons in Ireland, Dublin, IRL; 2 Surgical Oncology, Federal Government Poly Clinic (Post Graduate Medical Institute), Islamabad, PAK; 3 Surgical Oncology, Maroof International Hospital, Islamabad, PAK

**Keywords:** juvenile polyposis syndrome, non-familial, rectal prolapse

## Abstract

Juvenile polyposis syndrome is a rare inherited disorder that afflicts the gastrointestinal system. It usually occurs as a result of gene mutations; to date, several gene mutations, including those involving the bone morphogenetic protein receptor type IA (BMPR1A) gene, have been implicated in heralding the onset of the ailment. The disease is characterized by the infiltration of the gastrointestinal system with numerous hamartomas, which are predominantly benign. However, if left untreated, the hamartomas can undergo malignant transformations. Timely diagnosis and prompt surgical intervention are, therefore, imperative in portending favorable disease outcomes. We hereby delineate the case of a patient who presented with rectal prolapse and bleeding per rectum. Further diagnostic workup revealed the presence of polyps throughout the colon and the rectum, thereby insinuating a diagnosis of non-familial juvenile polyposis syndrome. The patient was managed through open surgery and continues to do well with no indications of disease recurrence.

## Introduction

Juvenile polyposis syndrome (JPS) is an autosomal dominant hereditary condition that occurs due to SMAD4 or bone morphogenetic protein receptor type IA (BMPR1A) gene mutations. It is characterized by the presence of multiple hamartomatous polyps in the gastrointestinal tract. While these polyps most typically present in the colon and rectum, they might also invade the stomach and parts or entirety of the small bowel [1]. JPS is inherently a benign condition; however, there is a 39% risk of progression to carcinoma without appropriate treatment [1, 2]. Notably, the estimated incidence of JPS is one case per 1,000,000 every year, with an average age at presentation hovering at nine years [2]. However, non-familial JPS remains an even rarer pathology and encompasses novel mutations and unremarkable family history. The clinical manifestations of JPS include anemia, alterations in bowel habits, and bleeding per rectum, with the age at presentation and the duration of symptoms differentiating JPS from other sinister pathologies. Nevertheless, its presentation as rectal prolapse in the absence of other symptoms is exceedingly rare [2, 3]. Herein, we chronicle a rare case of a 10-year-old male patient who presented with a history of lower abdominal pain and rectal prolapse. Clinical examination revealed diffuse infiltration of the rectum with polyps, alluding to the possibility of JPS. A further diagnostic evaluation revealed the presence of polyps throughout the colon and rectum. Considering the significant involvement of the gastrointestinal tract, a proctocolectomy with end ileostomy was performed. The patient continues to do well to date, with a regular screening at follow-up visits demonstrating complete disease remission.

## Case presentation

**Figure 1:**
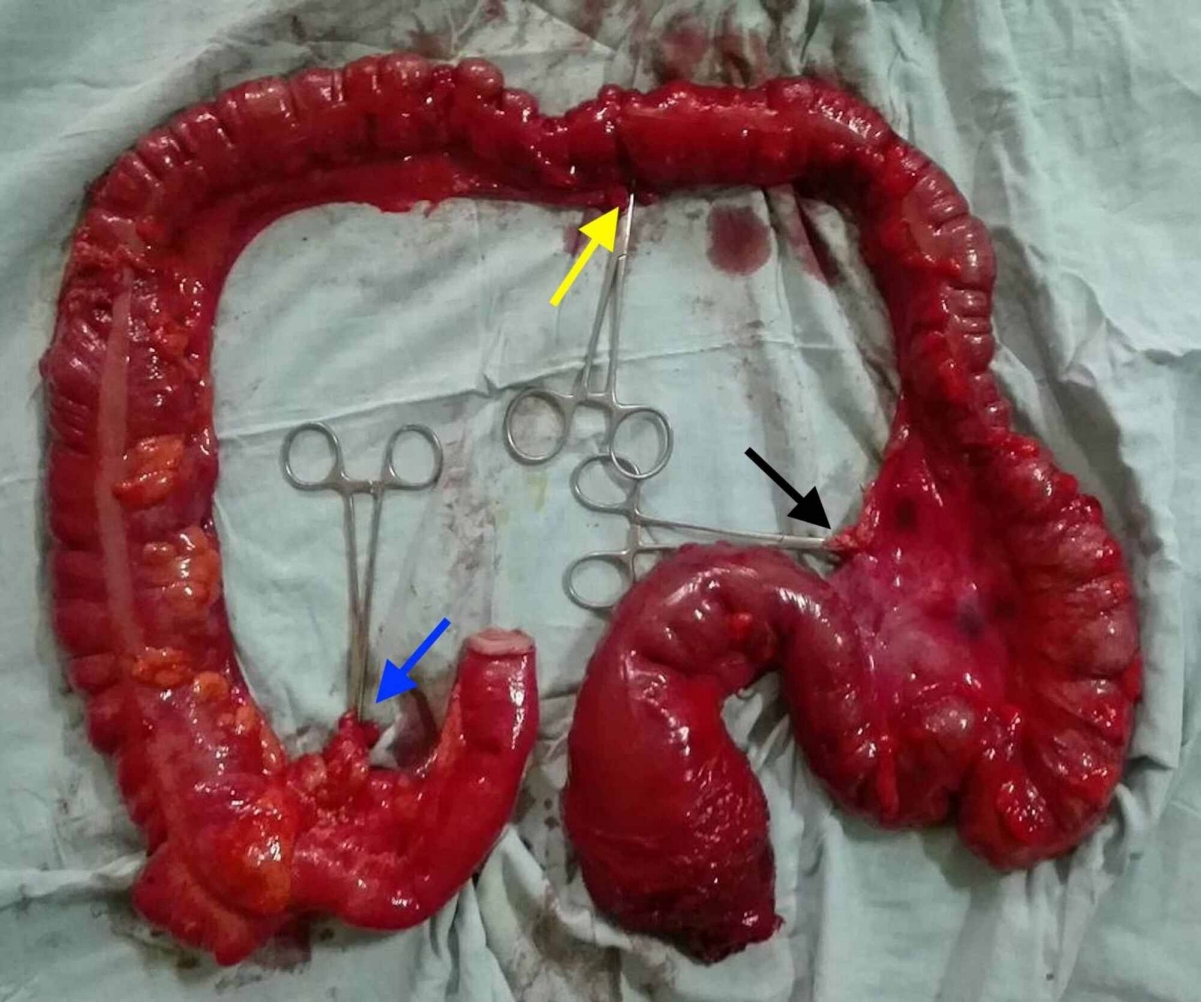
The excised specimen showing the distal ileum, cecum, ascending, transverse, descending, and the sigmoid colon along with the rectum Clips can be seen securing the ileocolic (blue arrow), middle colic (yellow arrow), and IMA (black arrow) pedicles. IMA - inferior mesenteric artery

We delineate the case of a 10-year-old boy who presented to the clinic with rectal prolapse and lower abdominal pain for the past one month. Pertinently, these symptoms were on a background history of the passage of frank blood and mucus per rectum for the past three weeks. Prior to the current presentation, the patient had not had any gastrointestinal complaints. Upon presentation to the clinical, the patient appeared well and in no obvious distress. Clinical examination revealed a full-thickness rectal prolapse, with the rectum replete with numerous distinct polyps across its entire diameter. Notably, there was no prior history of malena. Of the polyps observed, the lowest was noted to be three centimeters from the anal verge. Thereafter, a colonoscopy was performed and divulged a rectum completely infiltrated with polyps. In addition to the rectum, the aforesaid polyps were also observed along the length of the colon. Subsequent histopathological analysis revealed numerous pedunculated polyps in the colorectum and the terminal ileum. Sections of the polyps, including the multilobed forms, revealed ulcerated surface with underlying cystically dilated glands lined by columnar epithelium. The polyps were also observed to be budding and branching, admixed with adenomatous glands lined by dysplastic columnar epithelium. These findings insinuated an underlying diagnosis of JPS. Interestingly, the patient's family history was unremarkable for any gastrointestinal pathologies, including familial adenomatous polyposis (FAP). Therefore, the patient's disease was considered non-familial in nature. Considering the remarkable risk of progression to malignant transformation, a proctocolectomy with end ileostomy was performed, leaving behind three centimeters of the anal canal. The gross morphology of the excised specimen is delineated in Figure 1. 

**Figure 2:**
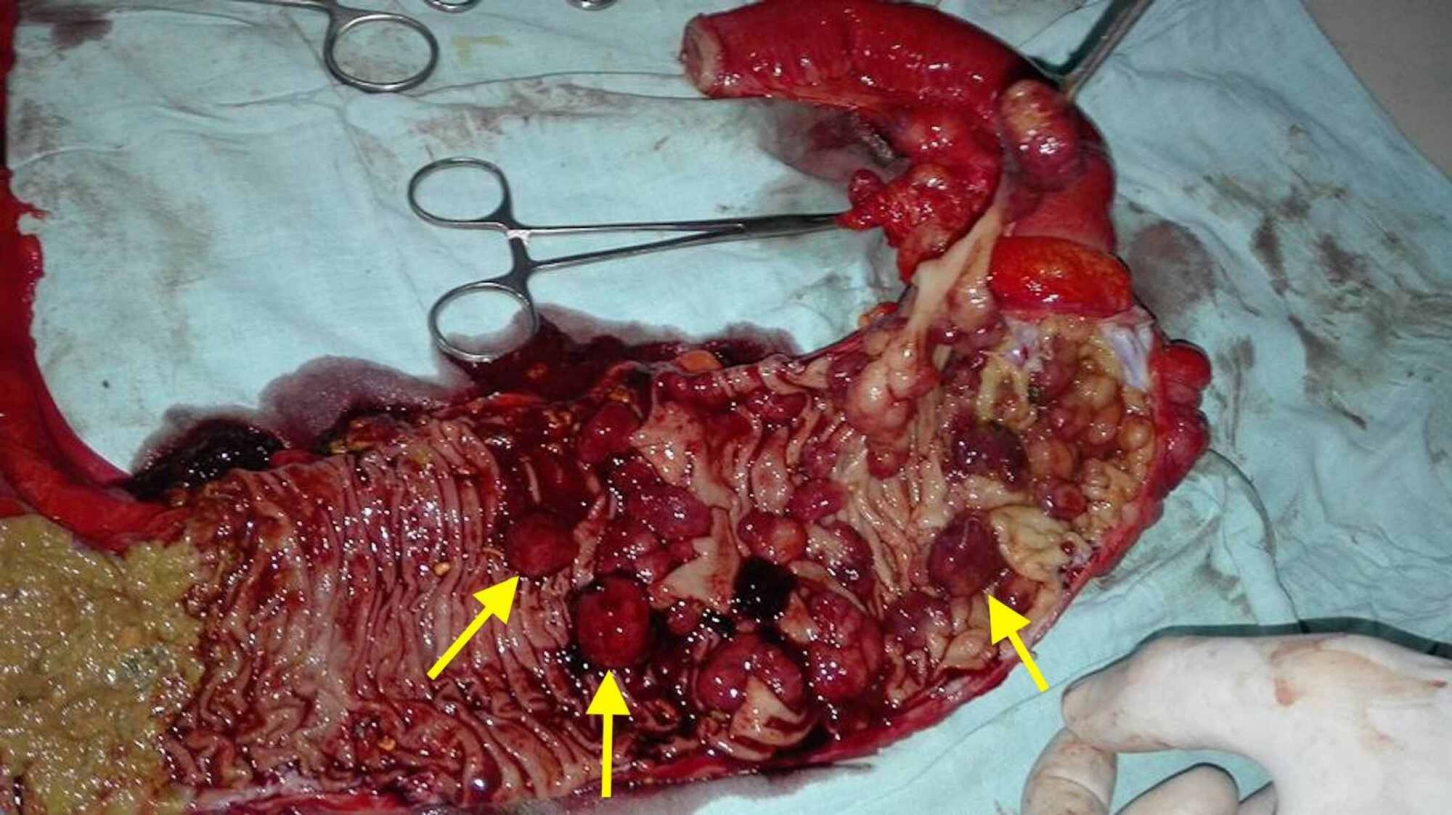
Resected cecum demonstrating multiple polyps (yellow arrows)

In order to better elucidate the etiology underlying the patient's symptoms, the excised cecum was resected. Diffuse infiltration with numerous polyps was seen (Figure 2). 

During the procedure, two liters of blood were transfused in order to maintain the patient's hemodynamic stability. There were no other intraoperative complications observed. The patient had an unremarkable postoperative recovery and was discharged in a stable condition. The patient continues to thrive, with regular follow-up visits showing no signs of disease recurrence and complete remission of symptoms. 

## Discussion

JPS remains an exceedingly rare hamartomatous polyposis syndrome, boasting a mere incidence of one in 100,000 [4]. JPS is defined by the occurrence of five or more hamartomatous polyps, either presenting at one time or recurrent, within any region of the gastrointestinal tract [5]. JPS is usually associated with mutations in either of the two genes; namely, the genes usually implicated are BMPR1A and SMAD4, which have been linked to the TGF-B/BMP signaling pathway. The pathology can either be sporadic or familial, with an autosomal dominant inheritance pattern [4]. Nevertheless, non-familial JPS, as observed in our case, remains starkly rarer than its inherited counterpart. Patients with JPS are at an increased risk of developing malignancies, with a 16% risk of colorectal carcinoma development in young patients and cumulative risk of 68% by 60 years of age [5].

Clinically, JPS usually presents as abdominal pain, diarrhea, bleeding per rectum, and anemia. Other less common and unusual presentations include intussusception and intestinal obstruction; however, its presentation as rectal prolapse is seldom encountered. The diagnosis of JPS requires an upper and lower tract endoscopy, polyp excision, and histopathological analysis. The diagnosis is based on the Jass criteria, which encompasses a plethora of features, such as the number of juvenile polyps found in one or more family members, polyps in colon or rectum greater than five in number, or juvenile polyps throughout the gastrointestinal tract [6]. We delineate a rare case of a 10-year-old boy who presented with rectal bleeding and rectal prolapse on a background of unremarkable family history, thereby alluding to the condition's non-hereditary nature. In order to effectively screen for JPS, upper endoscopy or colonoscopy should be performed to substantiate or refute any underlying suspicion. In our case, clinical examination revealed numerous polyps in the rectum, and a subsequent colonoscopy demonstrated numerous polyps throughout the colorectum, reaffirming the initial diagnosis of JPS.

Due to the malignant potential of the polyps, surgical treatment options are often warranted. The surgical treatment of choice in symptomatic patients with JPS involves surgical colectomy with ileorectal anastomosis, thus preserving the rectum [7]. Due to the extensive involvement of the entire colon and rectum in our case, a proctocolectomy with ileoanal anastomosis was performed. While more conservative surgical treatment options are available, the extensive colorectal involvement warranted the uptake of an invasive procedure in our case. The patient remains well to date, with complete remission of his symptoms. Therefore, prompt diagnosis and surgical management of the ailment remain pivotal in curbing the risk of malignant transformation in the future. 

## Conclusions

Non-familial JPS is an exceedingly rare pathology that elicits a myriad of clinical symptoms, including bleeding per rectum and anemia. However, its presentation as rectal prolapse is unusual, with meticulous clinical examination, including a digital rectal examination, often yielding the initial diagnostic clues. Physicians should remain cognizant of rectal prolapse as a potential clinical presentation of JPS in order to yield a prompt diagnosis and institute appropriate surgical treatment. 
